# Low muscle strength *vs.* low muscle mass for screening sarcopenia in patients undergoing hemodialysis

**DOI:** 10.1080/0886022X.2022.2156353

**Published:** 2023-01-12

**Authors:** Takahiro Yajima

**Affiliations:** Department of Nephrology, Matsunami General Hospital, 185-1 Dendai, Kasamatsu, Gifu 501-6062, Japan

Dear Editor,

I have read with great interest the paper written by Tian et al. [1] which was recently published in the RENAL FAILURE. They examined the associations of modified creatinine index (mCI) with ‘probable’ sarcopenia and mortality in patients receiving hemodialysis. They demonstrated that the mCI was a useful screening tool for sarcopenia and was a predictor of all-cause mortality in this population. The article was interesting; however, I have a few concerns and comments.

First, Tian et al. obtained the cutoff values of the mCI for detecting sarcopenia in men and women, respectively. However, the mCI is a powerful predictor which has already included sex [2], therefore, the cutoff value for predicting sarcopenia must be analyzed in overall patients. Furthermore, survival analyses should be performed using the cutoff point.

Second, they examined the relationships between the mCI and handgrip strength to investigate whether the mCI can be used as a tool for ‘probable’ sarcopenia or a tool for ‘screening’ sarcopenia. While, the mCI originally emerged as a surrogate maker of muscle mass volume [2]. According to the Asian Working Group for Sarcopenia: 2019 Consensus Update on Sarcopenia Diagnosis and Treatment, sarcopenia is defined as both low muscle strength and low muscle mass; indeed, compared with low muscle mass, low muscle strength is considered to be important [3]. However, in hemodialysis patients, Bataille et al. reported that the assessment of muscle mass volume is clinically important to diagnose sarcopenia, because muscle strength and muscle function are generally deteriorated [4]. In addition, Kim et al. and I showed that a loss of muscle mass, estimated by bio-impedance analysis, is independently associated with an increased risk of mortality in the Asian hemodialysis population [5,6]. Moreover, I have recently demonstrated that computed tomography-measured sarcopenic indices measured at the third lumber vertebra were also independently associated with increased risks of all-cause mortality [7–9]. These situations lead me to believe that low muscle mass should not be ignored in assessing sarcopenia in patients undergoing hemodialysis. Therefore, I recommend Tian et al. to reveal the diagnostic value of mCI for low muscle mass and sarcopenia and obtain cutoff values, and thereafter to evaluate the predictive value of mortality with the derived cutoff values.
